# Letter from the Editor in Chief

**DOI:** 10.19102/icrm.2025.16125

**Published:** 2025-12-15

**Authors:** Devi Nair


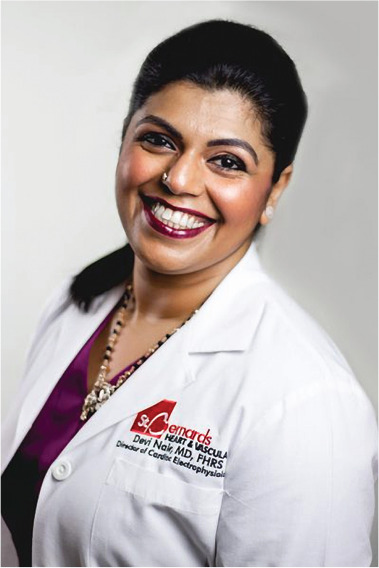


Dear Colleagues,

As we approach the close of another year, December offers a natural moment for reflection—an opportunity to look back on the progress we have made and ahead to the questions that continue to inspire our field. Over the past year, cardiac rhythm management has advanced at a remarkable pace, with innovations spanning ablation technologies, device therapy, imaging, and education. Through the pages of *The Journal of Innovations in Cardiac Rhythm Management*, we have had the privilege of sharing these developments and fostering a global dialogue focused on improving patient outcomes and deepening our understanding of complex arrhythmias.

## 2025: A Year of Momentum and Maturation

In 2025, several themes became increasingly clear. Technologies that once represented early promise continued to mature into practical, evidence-driven tools. Advances in ablation energy sources, device diagnostics, and physiologic pacing strategies refined how we approach arrhythmia care, shifting the focus toward greater precision, durability, and patient-centered decision-making.

Equally notable was the growing emphasis on integration. Imaging, electrophysiologic mapping, and device-based data increasingly converged to inform procedural planning and long-term management. This evolution reflects a broader movement within electrophysiology, one that values not only innovation but also thoughtful application grounded in anatomy, physiology, and clinical context.

## A Year of Discovery, Innovation, and Connection

Throughout the year, the way we learn and collaborate continued to evolve. International congresses, regional meetings, and virtual platforms strengthened connections across continents, allowing ideas and experience to be shared more rapidly than ever before.

The Rhythm Interventions Online Congress (RIO Congress), held virtually in November, exemplified this transformation. By leveraging a digital format, RIO brought together a truly global audience for high-quality, case-based education focused on real-world rhythm interventions. The Congress highlighted evolving ablation strategies, device therapy, and practical clinical decision-making while reinforcing how virtual platforms can expand access, promote inclusivity, and accelerate the translation of innovation into daily practice.

In many ways, RIO reflected the broader trajectory of our field, one that values collaboration without borders, embraces new educational models, and recognizes that meaningful progress occurs when knowledge is shared openly and widely.

## December Issue Highlights

The December issue reflects both the technical sophistication and thoughtful clinical insight that have characterized this year:

- Prakash et al. demonstrate that atrial electrogram–enabled single-chamber implantable cardioverter-defibrillators significantly improve detection of new-onset and asymptomatic atrial fibrillation, underscoring the expanding diagnostic role of contemporary device platforms.^1^
- In a compelling Images in Cardiac EP case, Ozeke et al. explore tachycardia termination without global propagation, offering a nuanced perspective on entrainment and circuit localization and reminding us of the enduring importance of fundamental electrophysiology principles.^2^
- Dasgupta and Johnsrude present instructive pediatric cases in which left-sided accessory pathways were successfully ablated from within the coronary sinus in patients with persistent left superior vena cava, highlighting the value of anatomic insight and procedural adaptability.^3^
- Weber and colleagues provide a state-of-the-art review of cardiovascular laser ablation, outlining its biophysical advantages and potential role as an alternative energy source in future rhythm interventions.^4^

Together, these contributions capture the breadth of modern electrophysiology, spanning devices, ablation, imaging, and mechanistic understanding, while maintaining a clear focus on translating innovation into meaningful clinical benefit.

## Looking Ahead

As we prepare to welcome a new year, the horizon remains filled with promise. Continued refinement of ablation technologies, expanding applications of physiologic pacing, and deeper integration of imaging and digital tools all hold the potential to further transform our practice. At the same time, important questions about durability, safety, access, and how best to individualize therapy for diverse patient populations remain.

Our Journal will continue to serve as a platform for addressing these challenges, sharing critical discoveries, and fostering the global collaboration that drives progress in cardiac rhythm management.

## A Note of Gratitude

As this year comes to a close, I would like to extend my deepest thanks to the authors, reviewers, and readers who make this Journal possible. Your commitment to scientific rigor, thoughtful inquiry, and collaborative exchange is the foundation upon which our field continues to advance. Each manuscript submitted, each review completed, and each article read contributes to a shared mission, improving the care of patients with cardiac arrhythmias worldwide.

I am especially grateful to our reviewers, whose dedication and expertise ensure the quality, integrity, and relevance of the work we publish, often through unseen and uncompensated efforts. To our authors, thank you for trusting this Journal as a platform for your ideas, innovations, and experiences. And to our readers, your engagement and curiosity drive the conversations that give meaning to these pages.

It is a privilege to serve this community. As we look ahead to the new year, I remain inspired by the collective spirit of discovery, mentorship, and collaboration that defines cardiac electrophysiology. I look forward to continuing this journey together, advancing knowledge, refining practice, and ultimately improving the lives of our patients.

Wishing you and your families a joyful holiday season and a healthy, fulfilling New Year.

Warm regards,


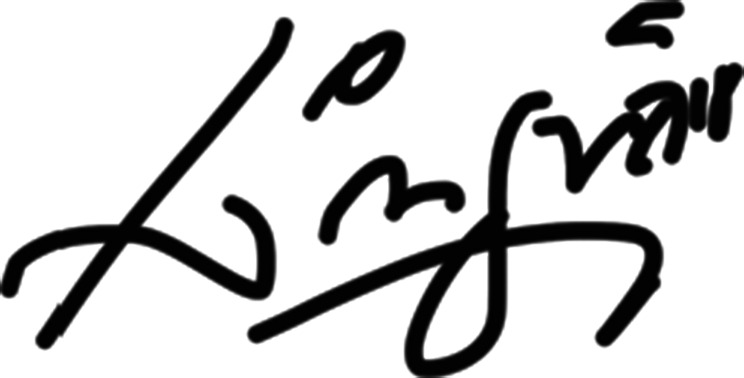


Dr. Devi Nair, md, facc, fhrs

Editor-in-Chief

*The Journal of Innovations in Cardiac Rhythm Management*

Director of the Cardiac Electrophysiology & Research,

St. Bernard’s Heart & Vascular Center, Jonesboro, AR, USA

White River Medical Center, Batesville, AR, USA

President/CEO, Arrhythmia Research Group

Clinical Adjunct Professor, University of Arkansas for Medical Sciences

Governor, Arkansas Chapter of the American College of Cardiology

drdgnair@gmail.com
